# Essential Role of *Sptan1* in Cochlear Hair Cell Morphology and Function Via Focal Adhesion Signaling

**DOI:** 10.1007/s12035-021-02551-2

**Published:** 2021-10-27

**Authors:** Qingxiu Yao, Hui Wang, Hengchao Chen, Zhuangzhuang Li, Yumeng Jiang, Zhipeng Li, Jiping Wang, Yazhi Xing, Feng Liu, Dongzhen Yu, Shankai Yin

**Affiliations:** 1 Department of Otolaryngology-Head and Neck Surgery, Shanghai Jiao Tong University Affiliated Sixth People’s Hospital, Shanghai, 200233 China; 2 Otolaryngology Institute of Shanghai Jiao Tong University, Shanghai, 200233 China; 3 Shanghai Key Laboratory of Sleep Disordered Breathing, Shanghai, 200233 China; 4 ENT Institute and Otorhinolaryngology Department, Affiliated Eye and ENT Hospital, Fudan University, Shanghai, 200031 China; 5 The First Affiliated Hospital, Zhejiang University School of Medicine, Hangzhou, 310003 China

**Keywords:** *Sptan1*, αII spectrin, Deafness, Stereocilia, Hair cell

## Abstract

Hearing loss is the most common human sensory deficit. Hearing relies on stereocilia, inserted into the cuticular plate of hair cells (HCs), where they play an important role in the perception of sound and its transmission. Although numerous genes have been associated with hearing loss, the function of many hair cell genes has yet to be elucidated. Herein, we focused on nonerythroid spectrin αII (SPTAN1), abundant in the cuticular plate, surrounding the rootlets of stereocilia and along the plasma membrane. Interestingly, mice with HC-specific *Sptan1* knockout exhibited rapid deafness, abnormal formation of stereocilia and cuticular plates, and loss of HCs from middle and apical turns of the cochlea during early postnatal stages. Additionally, *Sptan1* deficiency led to the decreased spreading of House Ear Institute-Organ of Corti 1 cells, and induced abnormal formation of focal adhesions and integrin signaling in mouse HCs. Altogether, our findings highlight SPTAN1 as a critical molecule for HC stereocilia morphology and auditory function via regulation of focal adhesion signaling.

**Supplementary Information:**

The online version contains supplementary material available at 10.1007/s12035-021-02551-2.

## Introduction

Hearing loss is the most common sensory disorder worldwide with genetic factors contributing to approximately half of congenital hearing loss cases [1]. Sensory epithelial hair cells, including the inner hair cells (IHCs) and outer hair cells (OHCs), contribute to normal hearing and balance via the conversion of movements initiated by sound waves into electrical signals [2–4]. Stereocilia, located on the top of hair cells (HCs), are microvillus-like protrusions containing actin filaments (F-actin) [5–7]. The rootlets of stereocilia insert into the dense F-actin mesh known as the cuticular plate, speculated to strengthen and provide additional anchoring for the stereocilia [7–9]. Previous studies have shown that the cuticular plate is enriched in various proteins, including myosin VI, which play important roles in the development and/or maintenance of stereocilia [10–16]. In our previous study, we found that myosin VI interacted with nonerythroid α II-spectrin (SPTAN1) [17], also known as alpha fodrin, encoded by *Sptan1*. Of note, SPTAN1 is distributed in the cuticular plate and along the lateral wall in HCs [18]; therefore, we speculated that *Sptan1* may play a critical role in HCs.

SPTAN1 is an essential cytoskeletal protein that ensures vital cellular properties, including polarity and cell stabilization. In addition, it is involved in cell adhesion, intercellular contact, and apoptosis [19]. Previous studies have demonstrated that *Sptan1* is required for the initial assembly of the axon segment, as well as for neuronal excitability, cortical lamination, and neuroprotection [20]. Additionally, Mahendrasingam et al. reported that SPTAN1 is present in the cortical lattice of OHCs [11], and Legendre et al. reported that SPTAN1 is widely distributed in auditory HCs, supporting cells, and fibroblasts [18]. Of note, SPTAN1 is not only distributed along the lateral wall of HCs, but also enriched in the cuticular plate [18]. Interestingly, Liu et al. reported that spectrin forms ring-like structures around the base of stereocilia rootlets and that HC-specific βII-spectrin knockout mice display disrupted HCs polarity and profound deafness [21]. However, the role of SPTAN1 in HCs remains unknown.

In this study, we developed an HC-specific *Sptan1* knockout mouse model to study the function of *Sptan1* in HCs. The knockout mice presented with improper bundle orientation and abnormal morphology of the cuticular plate, followed by HCs loss proceeding from apex to base and more pronounced in OHCs, which resulted in early-onset deafness. In addition, *Sptan1* knockdown in House Ear Institute-Organ of Corti 1 (HEI-OC1) cells led to abnormal actin distribution and decreased cell spreading. Importantly, the deficiency of *Sptan1* may induce the abnormal formation of focal adhesions and integrin signaling in both HEI-OC1 cells and mouse HCs. Our results suggest that *Sptan1* has a major role with respect to the morphology and function of HCs through the regulation of focal adhesion signaling.

## Materials and Methods

### Animals and Genotyping

*Sptan1* knockout mice were generated at the Nanjing Biomedical Research Institute of Nanjing University (Nanjing, China), using the clustered regularly interspaced short palindromic repeat (CRISPR)-associated Cas9 nuclease (CRISPR/Cas9) genome editing technique, as previously described [22, 23]. To generate a conditional allele for *Sptan1*, a targeting vector was designed to replace a 2.1 kb genomic fragment with loxP sites flanking exons 6, 7, and 8 of *Sptan1* (Fig. S1a). The guide RNA sequence used to target the locus with Cas9 was 5S5 GGCAGCTAAGTACGGCTCTTAGG and 3S1 AGCTAAGTCGAACAATTCAGTGG. This targeting vector construct was injected into oocytes of C57BL/6 J mice and the injected oocytes were implanted into foster female mice. The F0 generation was bred to obtain a F1 generation in which germ line transmission was confirmed. The F1 mice were genotyped using polymerase chain reaction (PCR) followed by sequence analysis. Upon crossing the mice with cochlear hair cell (HC)-specific Cre recombinase-expressing mice, exons 6–8 were excised, resulting in a short *Sptan1* transcript with a 218-amino acid N-terminal and one spectrin repeat. *Sptan1 *^*f/f*^ mice were crossed with *Gfi1-Cre* mice to produce cochlear HC-specific conditional knockout (CKO) mice (Fig. S1a). The background strains of *Sptan1* mice were C57BL/6 J. Heterozygous *Gfi1*^*cre/*+^ mice were generated in a 129S6 and C57BL/6 J mixed background as previously described, and the recombination efficiency of *Gfi1-Cre* was approximately 93% in HCs at postnatal day (P) 0 [24]. Male and female homozygous (*Gfi1-Cre*^*−/* +^;*Sptan1*^*f/f*^) animals (*Sptan1*-CKO group) and control siblings (*Gfi1-Cre*^*−/* +^;*Sptan1*^*f/*+^*)* (control group) were used in the present study. Mice were genotyped according to standard PCR protocols with extracted mouse tail tips [25]. The primer locations are shown in Fig. S1b. We identified *Sptan1* alleles using PCR primers (Table. S1), which detected *Sptan1*, *Sptan1* 3′, and *Sptan1* 5′. The *Sptan1*^*f/f*^ allele was targeted by the *f* and *r* primers and identified with a 300 bp band, whereas *Sptan1*^+*/*+^ was identified with a 211 bp band. *Sptan1*^*f/*+^ was identified through the simultaneous presence of 300 bp and 211 bp bands. *Sptan1*^*f/*+^ was also identified with a 1011 bp band when using a *Sptan1* 5′ primer and an 1171 bp band when using a *Sptan1* 3′ primer (Fig. S1c). The *Gfi*-1F and *Gfi*-1R primers were used to identify the wildtype allele (609 bp), and *Gfi*-1F and *Gfi1*Cre-R to identify the mutant allele (672 bp).

Both *Sptan1*-CKO and control groups contained mice with equal proportions of males and females. Animals were housed in individually ventilated cages. The temperature inside the cages was maintained at 25 °C with a relative humidity of 40–70% and 60–65 air changes/hour in the cage. The cages including the bedding and nesting material were changed weekly. Feed and water were autoclaved prior to use.

### Cell Culture and Transfection

The HEI-OC1 cell line (House Ear Institute, Los Angeles, USA), an inner ear cell line derived from the Immortomouse, expresses markers for auditory sensory cells, and is widely used to study the function of cochlear HCs. HEI-OC1 cells were cultured in high-glucose Dulbecco’s modified Eagle’s medium (Gibco, Grand Island, MD, USA) supplemented with 10% fetal bovine serum (FBS; Gibco) and 50 μg/mL ampicillin (Sangon Biotech, Shanghai, China) at 33 °C under an atmosphere of 10% CO_2_. Knockdown of *Sptan1* was performed using a lentivirus expressing either shRNA targeting *Sptan1* or non-relevant shRNA as a control (Hanbio Biotechnology, Shanghai, China), according to the manufacturer’s instructions. The targeted sequences were as follows: *Sptan1* shRNA: 5′-CATAACTAAGGAGGCCGGCAGTGTA-3′; non-relevant shRNA: 5′-TTCTCCGAACGTGTCACGTAA-3′. Briefly, 2 µL shRNA was premixed with 5 × 10^3^ cells during the submerged culture stage. Puromycin selection was performed to eliminate untransfected cells 24 h after transfection. The transfected cells simultaneously expressed green fluorescent protein (GFP) as a marker. We performed western blotting to verify transfection efficiency.

### Auditory Physiological Assessment

Auditory brainstem responses (ABRs), distortion product otoacoustic emissions (DPOAEs), and compound action potentials (CAPs) were recorded using the Tucker–Davis hardware and BioSig software (Tucker–Davis Technology System III; TDT, Alachua, FL, USA) as previously described [26]. In total, 24 male and female mice at P30 and eight at P22 underwent auditory physiological testing. The ABR was recorded first, followed by DPOAE and CAP recordings. For all physiological tests, mice were anesthetized with intraperitoneal injections of pentobarbital sodium (50 mg/kg body weight). Mouse body temperature was maintained at 37 °C with a thermostatic heating pad during testing and recovery from anesthesia.

For the ABR recordings, tone bursts (1 ms rise/fall with an 8 ms plateau from 4 to 24 kHz at a rate of 21.1 /s) of stimuli were presented through a broadband speaker (FostexFT28D Dome Tweeter, Madisound, Madison, WI, USA) placed 10 cm in front of the mice. Evoked responses were recorded using three subdermal electrodes with the recording electrode inserted at the vertex of the skull and reference and grounding electrodes behind both pinnae. The signal was amplified 20 × , filtered between 300 and 3000 Hz with a PA16 preamplifier (TDT), and averaged over 1000 repetitions using BioSig software. The stimulus decreased from 105 to 0 dB sound pressure level (SPL) in 5-dB steps, and the threshold of response was judged as the lowest SPL at which a repeatable wave III was visible. If no waveform was identified at the highest presentation level (105 dB SPL) for a particular frequency, the threshold was recorded as 110 dB SPL.

For DPOAE measurements, two MF1 speakers (TDT) and a microphone were used to present two pure-tone stimuli and collect the distorted product, respectively, from the tested outer ear canal via a 10 cm length of plastic tubing. Two pure-tone stimuli at frequencies f1 and f2, with a frequency ratio (f2/f1) of 1.2 and the f2 level 10 dB lower than the f1 level, were generated and delivered simultaneously from 80 to 0 dB in 5-dB steps. The f2 frequency was swept from 2.8 to 22.6 kHz with an increase of 1/2 octaves. The distorted product was amplified by 40 dB with an ER-10 B + low noise mic system (Etymotic Research, Inc., Elk Grove, IL, USA) before outputting to the TDT system. The DPOAE threshold was considered the lowest level of f1 that corresponded to a peak at 2f1–f2 that was at least 6 dB above the floor of the noise surrounding 2f1–f2.

The same stimuli were used for ABR and CAP recordings. CAPs were recorded using a silver ball electrode at the round window after surgically opening the mastoid. The grounding and reference electrodes were placed around the incision. Evoked responses were amplified using a PA16 preamplifier for CAPs (filtered below 3 kHz) before being averaged 100 times. The CAP amplitude was measured between the largest negative peak (N1) and the following positive peak (P1). This surgical procedure received ethical approval.

### Immunofluorescence Staining

Immunofluorescence (IF) staining was performed on whole-mount sections, cryostat sections of the inner ears, and HEI-OC1 cells. The inner ears were prepared for immunostaining using a previously described standard procedure [27]. Briefly, the inner ears were rapidly removed with the round and oval windows opened and a hole made at the apex, fixed overnight in 4% paraformaldehyde (PFA), and then decalcified in 10% EDTA for 1 day. For whole-mount sections, the organ of Corti was dissected. For cryosections, the inner ears were dehydrated with sucrose, embedded in OCT medium, and cut into 10-μm sections. HEI-OC1 cells grown on glass coverslips (WHB Scientific, Shanghai, China) were washed in PBS and fixed in 4% PFA. The organ of Corti and cells were permeabilized with 0.5% Triton-X 100, blocked in staining blocking buffer (Beyotime Biotech, Shanghai, China), and incubated with primary antibody targeting alpha fodrin (1:200, D8B7, Abcam, Cambridge, UK), SPTBN1 (1:200, BD Biosciences, San Jose, CA, USA), myosin VIIa (1:500, Proteus BioSciences, Ramona, CA, USA), myosin 6, TRIOBP, FAK, Integrin β1, TALIN, VINCULIN and PAXILLIN (1:200, ProteinTech Group, Chicago, USA), cleaved-caspase 3 (1:200, Cell Signaling Technology, Boston, USA) overnight at 4 °C. On the second day, they were washed and incubated with Alexa Fluor® 488 goat anti-mouse IgG1 (γ1) (1:500 Invitrogen, Carlsbad, CA, USA), Alexa Fluor® 647 goat anti-mouse IgG2b (γ2b) (1:500, Invitrogen), Alexa Fluor® 633 goat anti-mouse (1:500, Invitrogen), or Alexa Fluor® 633 goat anti-rabbit (1:500, Abcam) secondary antibodies (Table. S3) for 1 h at room temperature. Subsequently, they were incubated with Phalloidin-iFluor 555 reagent (1:2000; Abcam, Cambridge, UK), counterstained with Fluoroshield with DAPI histology mounting medium (Sigma-Aldrich, St. Louis, MO, USA) or DAPI (5 μg/ml, Beyotime Biotech) and imaged using an LSM 710 confocal microscope (Zeiss, Oberkochen, Germany) at 20 × , 63 × or 100 × magnification. The images were processed and analyzed using the ZEN 2011 software (Zeiss) and the ImageJ Fiji software for calculating fluorescent intensities.

### Cochlear Hair Cell Count

For quantitative evaluation of cochlear HC loss in *Sptan1*-CKO and control groups, succinate dehydrogenase histochemistry (SDH) staining was performed as previously described [28]. Briefly, P15 and P30 mice were sacrificed under deep anesthesia; the inner ears were rapidly removed and gently perfused with freshly prepared SDH staining solution for 45 min at 37 °C, and then fixed in 4% PFA for 24 h at 4 °C. Whole-mount sections were prepared as described above, and the cells were examined under a light microscope (Nikon, Tokyo, Japan) at 40 × magnification. The numbers of both types of HCs were counted over 0.24 mm intervals from the apex to the base along the entire length of the cochlea. The data were input into a custom program, and the percentages of missing IHCs and OHCs were calculated as a function of percentage distance along the entire basilar membrane based on apex to base HC count norms for C57 mice [29]. Data from each individual cochleogram were averaged to generate a mean cochleogram for each experimental condition.

### Detection of Apoptosis Using Caspase-3

To explore the possible mechanism underlying HC apoptosis, we identified changes in the expression level of caspase-3. First, FITC-DEVD-FMK from the Caspase-3 Detection Kit (Invitrogen) was freshly prepared at a dilution of 1:100 and injected into the scala media. After 1 h, the cochleae were then washed three times with wash buffer and fixed with ice-cold 4% PFA in Hank’s balanced salt solution overnight. Other procedures, including cochleae decalcification and cochlear membrane microdissection, were performed as described above. The specimens were then stained with DAPI to label the nuclei. Finally, the specimens were examined and imaged using a confocal microscope at × 63 magnification.

### Western Blotting

Ten dissected cochlear tissues and HEI-OC1 cells were washed in PBS and harvested in lysis buffer (Beyotime Biotech) supplemented with Halt Protease Inhibitor Cocktail (Thermo Fisher Scientific, Waltham, MA, USA). Whole proteins were extracted and quantified using the Bicinchoninic Acid Protein Assay Kit (Thermo Fisher Scientific). A sample of total protein (30 μg) was mixed with 5 × SDS loading buffer, and proteins were separated using 7.5% SDS-PAGE (Beyotime Biotech). Proteins were transferred onto a PVDF membrane (Invitrogen) using a blotting system (Bio-Rad, Hercules, CA, USA). The membranes were blocked in 5% non-fat dry milk for 1 h at room temperature and incubated with primary antibodies against alpha fodrin (1:1000; Abcam), SPTBN1 (1:1000; BD Biosciences), beta-actin or GAPDH (1:1000; Invitrogen) overnight at 4 °C. On the second day, membranes were incubated with horseradish peroxidase (HRP)-conjugated secondary antibodies (1:1000, Beyotime Biotech) for 1 h at room temperature. The membranes were developed with enhanced electrochemiluminescence (ECL) solution (Beyotime Biotech), imaged using the ChemiDocXRS imaging system (Bio-Rad) and analyzed using the ImageJ software (NIH, Bethesda, MD, USA).

### Real-Time PCR

Total RNA was extracted from four dissected cochleae using TRIzol reagent (TaKaRa Bio, Kusatsu, Japan) according to the manufacturer’s instructions. The RNA concentration was determined using a NanoDrop 2000 c spectrophotometer (Thermo Fisher Scientific), and RNA integrity was evaluated using agarose gel electrophoresis and staining with ethidium bromide. RNA (1 μg) was reverse transcribed to cDNA using a Quantitect Reverse Transcription Kit (Qiagen, Hilden, Germany). Real-time PCR (RT-PCR) was performed using a LightCycler® 480 II Real-time PCR Instrument (Roche, Basel, Switzerland) with a 10 μL PCR mixture that included 1 μL of cDNA, 5 μL of 2 × QuantiFast® SYBR® Green PCR Master Mix (Qiagen), 0.2 μL of forward primer, 0.2 μL of reverse primer, and 3.6 μL of nuclease-free water. The reactions were incubated in a 384-well optical plate (Roche) for 5 min at 95 °C, followed by 40 cycles of 95 °C for 10 s and then 60 °C for 30 s. Each sample was run in triplicate for analysis. The primers were designed and synthesized by Generay Biotech (Shanghai, China) based on the mRNA sequence obtained from the NCBI database. All sequences of primers used for RT-PCR are shown in Table S2. *GAPDH* was used as an internal control. An identical threshold for fluorescence was applied for each gene of interest. Relative mRNA expression levels were calculated using the 2 ^−ΔΔCt^ method (threshold cycle (Ct)) and linearized Ct values before averaging [30].

### Scanning Electron Microscopy

The dissected inner ears were fixed in 2.5% glutaraldehyde in phosphate buffer by intralabyrinthine perfusion overnight at 4 °C. The tissues were then washed in 0.01 M PBS, dissected to reveal the organ of Corti, and post-fixed with 1% osmium tetroxide in phosphate buffer for 2 h at 4 °C. Post-fixation, the tissues were dehydrated with a graded series of ethanol and dried using an EM CPD 300 critical point dryer with liquid CO_2_ (Leica, Wetzlar, Germany). The dehydrated tissues were sputter-coated with platinum (15 nm) using an EM SCD 050 instrument (Leica) and then analyzed using a Quanta 250 field-emission scanning electron microscope (FEI, Brno, Czech Republic). Three specimens were examined in both the *Sptan1*-CKO group and the control group.

### Immunoprecipitation and Mass Spectrometry

Six cochleae of each group were chopped into small pieces and lysed on ice with lysis buffer (Servicebio, China) for 30 min. Twenty microliters protein A/G agarose beads (Millipore, Temecula, CA) were added into the supernatant for 30 min. After centrifugation, the pellet was discarded. The supernatant was immunoprecipitated using 2 μg antibodies against SPTAN1 (Abcam, Cambridge, UK) or SPTBN1 (BD Biosciences, San Jose, CA) overnight at 4 °C and pulled down using fully re-suspended protein A/G magnetic beads. The IgG was used as negative control. Samples were washed with lysis buffer and boiled, subjected to SDS-PAGE. Gels were stained with Coomassie Blue and protein bands were collected for mass spectrometry analysis. After reduction and alkylation, the samples were digested by Trypsin overnight at 37 °C, and analyzed by nLC-MS/MS using a Q-Exactive mass spectrometer (Thermo Fisher Scientific) coupled with an EASY-nLC 1000 (Thermo Fisher Scientific) chromatography system, which was carried out by Shanghai Applied Protein Technology (Shanghai, China).

### Statistical Analysis

Statistical analysis was performed using Sigma Plot (ver. 14.0; Systat Software Inc., San Jose, CA, USA). Normally distributed data were represented as mean ± standard error of the mean (SEM), while non-normally distributed data were represented as medians with the 25–75% interquartile range. One-way analysis of variance (ANOVA) followed by Tukey’s post hoc test for multiple comparisons were used to determine significant differences of *Sptan1* mRNA expression among groups. Two-way ANOVA were performed to determine significant differences in ABR, DPOAE, and CAP tests. The *χ*^2^ test was used to determine significant differences of the incidence of stereociliary abnormalities between *Sptan1*-CKO group and control group, and the percentage of rounded cell shapes between *Sptan1* depletion and negative control cells. Non-parametric test was used to determine relative quantification of SPTBN1 protein by intensity analysis. *P* values < 0.05 were considered statistically significant. Figures were generated using the Prism software (GraphPad Software, La Jolla, CA, USA).

## Results

### SPTAN1 Is an Abundant Component of the Cuticular Plate and Rootlets of Stereocilia

**Fig. 1:**
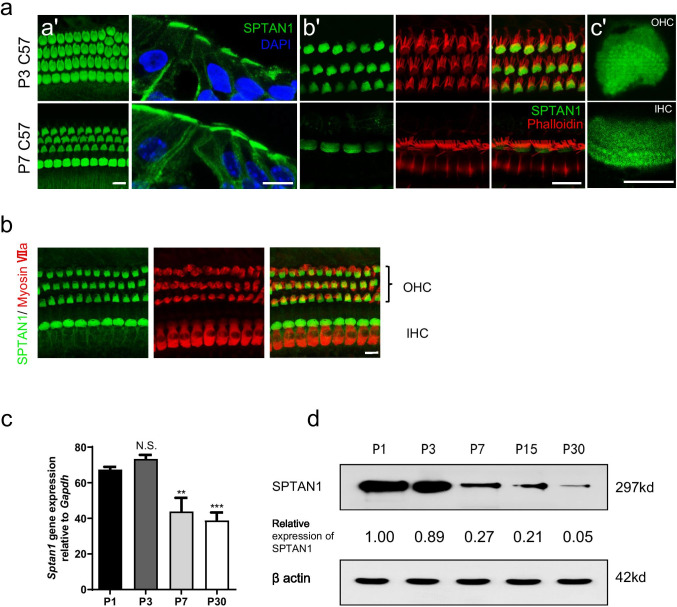
*Sptan1* is an abundant component of the cuticular plate and rootlets of stereocilia. **a** (a′) Immunofluorescence of SPTAN1 in whole-mount staining and cryostat sections of the cochleae of P3 and P7 C57 BL/6 J mice. Scale bar: 10 μm. (b′) Immunofluorescence of SPTAN1 in whole-mount staining in guinea pigs. SPTAN1 (green) is mainly expressed in the cuticular plate and along the plasma membrane of hair cells. Scale bar: 10 μm. (c′) SPTAN1 is expressed in rootlets of stereocilia of OHC and IHC. Scale bar: 2.5 μm. **b** SPTAN1 co-expresses with Myosin VIIa in hair cells in P15 mice. Scale bar: 10 μm. **c** RT-PCR analysis of *Sptan1* mRNA expression in the organ of Corti of C57BL/6 J mice (*n* = 6). The expression of *Sptan1* at P3 is similar to P1, and the expression at P7 and P30 is significantly lower than P1. One-way analysis of variance (ANOVA) followed by Tukey’s post hoc test for multiple comparisons were used to determine significant differences among groups. Error bars represent standard error of the mean. **P < 0.01, ***P < 0.001. **d** Analysis of SPTAN1 expression by western blotting showing higher levels at P1 and P3 than at P7, P15, and P30 in the organ of Corti

A previous study revealed that SPTAN1 is abundant in the cuticular plate [21]. To examine the spatiotemporal expression of *Sptan1* in normal cochlear HCs at various stages of development, we used immunofluorescence staining (IF), real-time PCR (RT-PCR), and western blotting. SPTAN1 was abundant both in the cuticular plates and along the plasma membranes of HCs (Fig. 1a (a′–b′)). In addition, strong SPTAN1 expression was observed around the rootlets of stereocilia, but not in the stereocilia (Fig. 1a (c′)). SPTAN1 was roughly equal expressed in apical, middle, and basal turns (Fig. S2a). Moreover, SPTAN1 was co-expressed with Myosin VIIa in HCs (Fig. 1b). In addition, it was still expressed in the cortical lattices of Deiters’ cells, pillar cells, and other supporting cells (Fig. S2b). Interestingly, the expression of *Sptan1* at both the mRNA and protein levels was decreased during development (Fig. 1c–d). In summary, our results show that HCs are enriched in SPTAN1, specifically localized in the cuticular plate and rootlets of stereocilia.

### *Sptan1*-CKO Mice Develop Hearing Loss

**Fig. 2:**
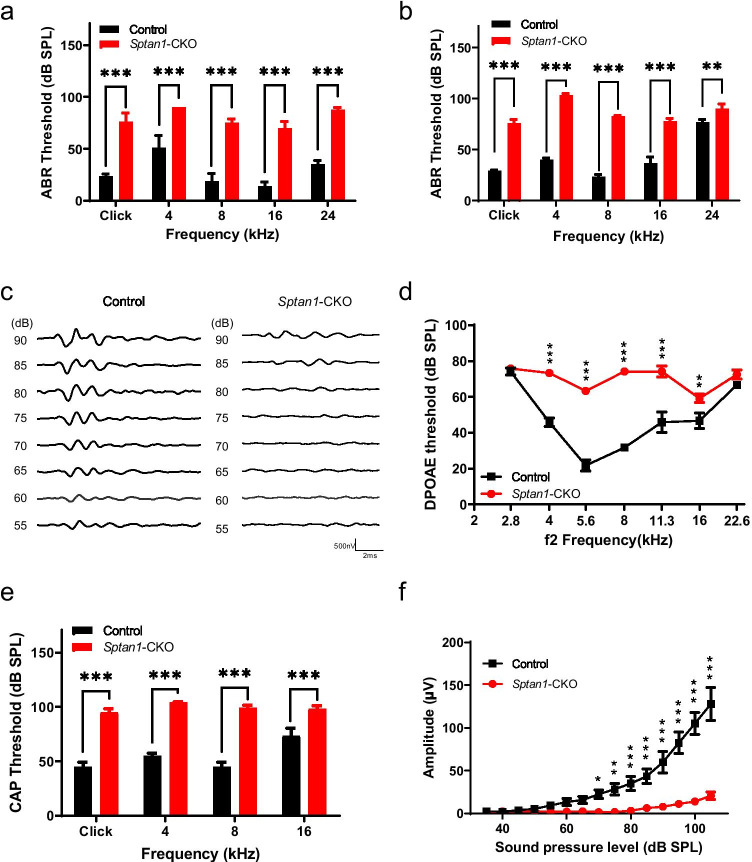
*Sptan1*-CKO mice at P22 and P30 show elevated hearing thresholds. **a** *Sptan1*-CKO mice show increased ABR thresholds (*n* = 4, red columns) compared to those of control mice (*n* = 4, black columns) at clicks and 4, 8, 16, 24 kHz at P22. **b** *Sptan1*-CKO mice show increased ABR thresholds (*n* = 6, red columns) compared to those of control mice (*n* = 6, black columns) at clicks and 4, 8, 16, 24 kHz at P30. **c** Representative ABR traces recorded from P30 control (*n* = 3) and *Sptan1*-CKO mice (*n* = 3), with an 8-kHz tone burst between 55- and 90-dB SPL. **d** DPOAE thresholds for pure-tone stimuli in *Sptan1*-CKO (*n* = 6, red line) are significantly higher than those in control animals except at 2.8 and 22.6 kHz at P30 (*n* = 6, black line). **e** *Sptan1*-CKO mice show higher CAP thresholds (*n* = 6, red columns) than control mice (*n* = 6, black columns) at P30. **e** *Sptan1*-CKO mice (*n* = 6, red line) show lower CAP amplitudes from 70 to 105 dB SPL at 8 kHz than control mice (*n* = 6, black line) at P30. The two-way ANOVA was used to determine significant differences in **a**, **b**, **d**, **e**, and **f**. Error bars represent standard error of the mean. *P < 0.05, **P < 0.01, ***P < 0.001

We examined the resulting auditory physiological properties, including the auditory brainstem responses (ABRs), distortion product otoacoustic emissions (DPOAEs), and compound action potentials (CAPs) in two groups. The ABR thresholds for clicks and 4, 8, 16, and 24 kHz at P22 were 76.25 ± 8.20, 90 ± 0, 75 ± 3.54, 70 ± 6.12, 87.5 ± 2.5 dB SPL in *Sptan1*-CKO mice (*n* = 4), respectively, compared to 23.75 ± 2.17, 51.25 ± 11.39, 18.75 ± 7.40, 13.75 ± 4.15, 35 ± 3.54 dB SPL in control mice (*n* = 4), respectively. The results showed significantly elevated thresholds at clicks and 4, 8, 16, and 24 kHz (*P* < 0.001) in *Sptan1*-CKO mice (Fig. 2a). The ABR thresholds for clicks and 4, 8, 16, and 24 kHz at P30 were 75.80 ± 8.86, 103.33 ± 3.73, 82.50 ± 2.50, 77.50 ± 7.50, and 90.00 ± 11.55 dB SPL in *Sptan1*-CKO mice (*n* = 6), respectively, compared to 29.17 ± 1.86, 40.00 ± 4.08, 23.33 ± 4.71, 36.67 ± 14.04, and 76.66 ± 6.87 dB SPL in control mice (*n* = 6), respectively. The results showed significantly elevated thresholds at clicks and 4, 8, 16 (*P* < 0.001), and 24 kHz (*P* < 0.01) in *Sptan1*-CKO mice (Fig. 2b). The ABR raw traces of the two groups at P30 are shown in Fig. 2c.

At P30, the DPOAE thresholds of *Sptan1*-CKO mice were also significantly increased compared to those of control mice at 4, 5.6, 8, 11.3 (*P* < 0.001), and 16 kHz (*P* < 0.01) (Fig. 2d). Similarly, the CAP thresholds were significantly increased (*P* < 0.001) (Fig. 2e), and the CAP amplitudes were significantly decreased from 70 to 105 dB SPL at 8 kHz in *Sptan1*-CKO versus control mice (*P* < 0.05) (Fig. 2f).

### HCs of *Sptan1*-CKO Mice Show Stereocilia and Cuticular Plate Defects

**Fig. 3:**
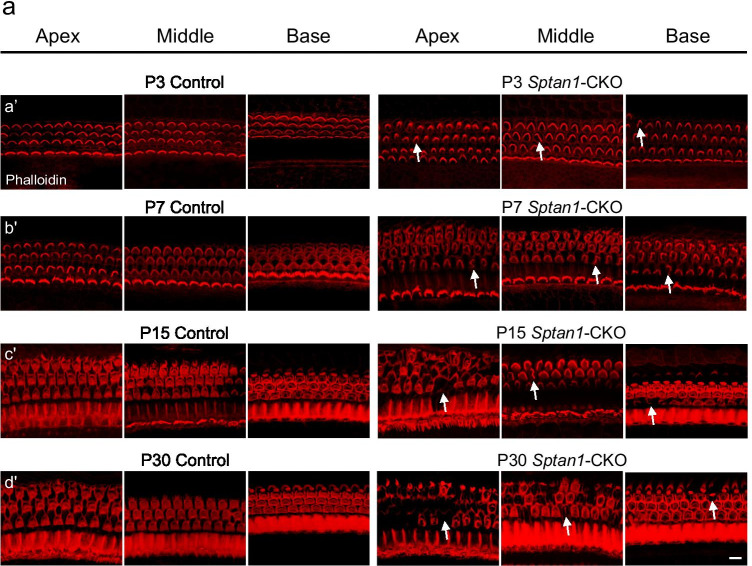

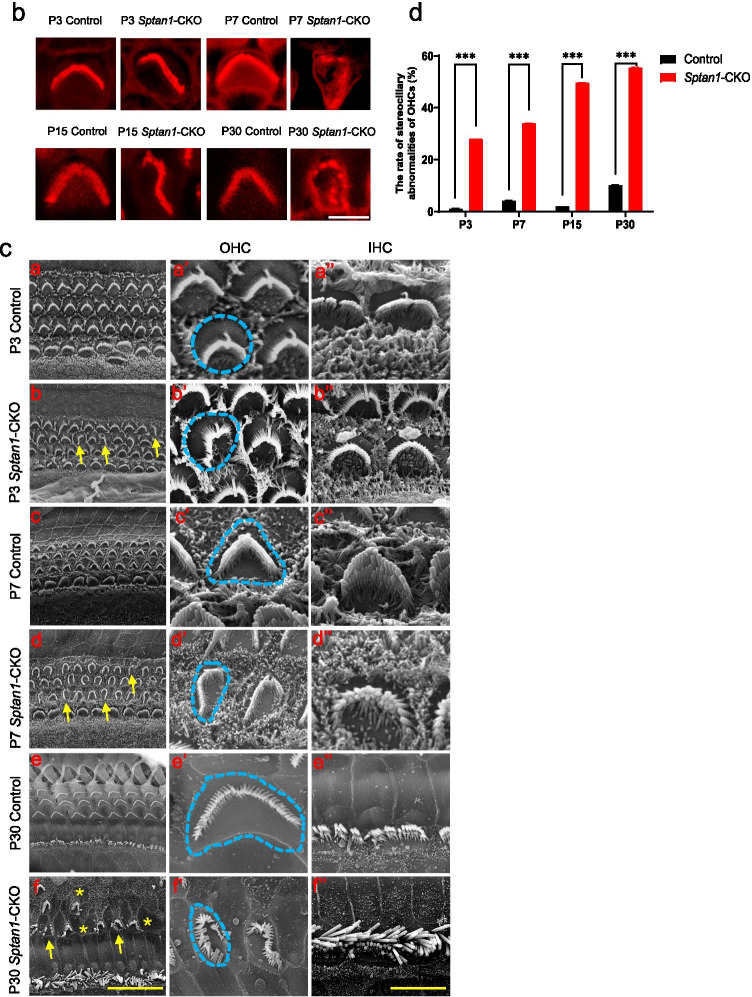
Outer hair cells (OHCs) of *Sptan1*-CKO mice exhibit severely altered stereocilia and cuticular plate morphologies. **a** (a′) At P3, in control mice, the OHCs show normal V-shaped hair bundles, whereas in *Sptan1-*CKO mice, some stereocilia exhibit “U” and “C” shapes or partially missing. (b′) At P7, there are more abnormal stereocilia than at P3 in *Sptan1-*CKO mice. (c′) At P15, some stereocilia show abnormal shapes in *Sptan1-*CKO mice and some even lost. (d′) At P30, the loss of hair bundles was more severe than at P15 in *Sptan1-*CKO mice. The loss of stereocilia was more severe in apical and middle regions than basal region. Abnormal stereocilia bundles are highlighted by arrows. Scale bar: 10 µm. **b** Higher magnification of stereocilia in OHCs. Scale bar: 5 µm. **c** (a–f) Low-magnification SEM images of cochlear hair bundles from mice of different genotypes and ages. The stereocilia of OHCs started to lose their polarity as early as at P3. The extent of the disruption is larger at P7. Some loss is apparent at P30. The abnormal shapes of stereociliary bundles are highlighted by yellow arrows, and stereocilia loss is indicated by asterisks. In Fig. 3cf, the stereocilium indicated by the left yellow arrow is significantly fused, without staircase architecture, compared to that of other stereocilia. Scale bar: 20 µm. (a′–f′) High-magnification SEM images of OHCs hair bundles from mice of different genotypes and ages. The shapes of cuticular plates became oval in *Sptan1-*CKO mice, as highlighted by the blue circles. (a″–f″) High-magnification SEM images of inner hair cells (IHCs) hair bundles from mice of different genotypes and ages. Images of different turns of the cochleae were captured. **a**, **b**, **c**, and **d** represent apical-middle turns, and **e** and **f** represent middle-basal turns. Scale bar: 5 μm. **d** The incidence of stereociliary abnormalities of OHCs throughout entire length of the cochlear basilar membrane at different ages is significantly higher in *Sptan1-*CKO versus control mice, as per the *χ*^2^ test. At each time point, ~ 600 OHCs from 3 mice in each group were analyzed. Error bars represent standard error of the mean. *** *P* < 0.001

**Fig. 4:**
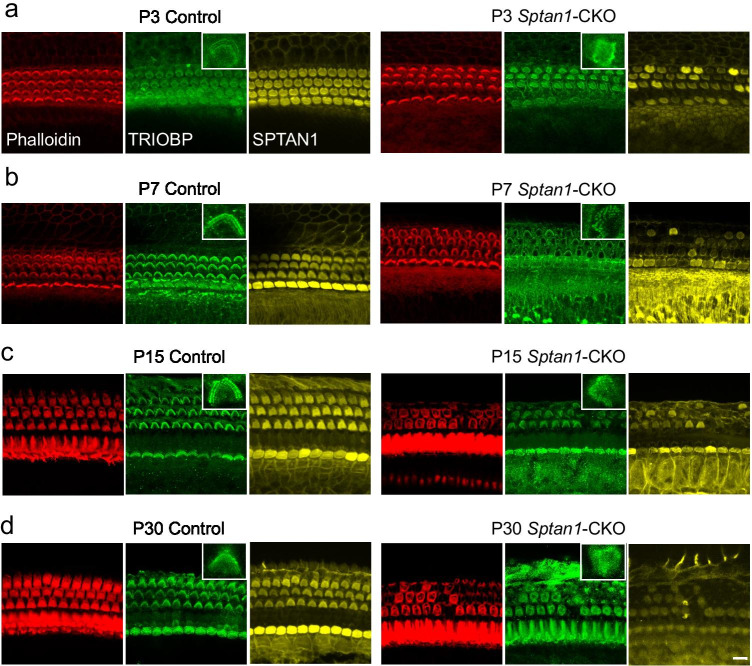
The rootlets of stereocilia are abnormal in *Sptan1*-CKO mice of different ages. The organ of Corti was stained with anti-TRIOBP (green) antibodies and Phalloidin (red). In the control group, the rootlets of stereocilia of OHCs are V-shaped. **a** At P3, in *Sptan1-*CKO mice, some rootlets of stereocilia of OHCs are deformed. **b** At P7, there are more misorientated rootlets of stereocilia of OHCs than at P3 in *Sptan1-*CKO mice. **c** At P15, abnormal rootlets of stereocilia of OHCs are increased in *Sptan1-*CKO mice; some degree of loss is also presented. **d** At P30, more rootlets of stereocilia loss is evident in *Sptan1-*CKO mice (*versus* P15). The rootlets of stereocilia of IHCs are also slightly disordered at P30. All images were captured in the middle turns. Scale bar: 10 µm

Next, we performed phalloidin staining, IF, and scanning electron microscopy (SEM) to investigate changes in the morphology of stereocilia at different regions and development stages. The orientation of each individual hair bundle was assessed by determining the rotation of the bundle relative to a line extending perpendicular to the row of pillar cells that separates the IHCs and OHCs. Cells with a normal orientation were aligned along this perpendicular axis and so have a rotation of 0°. Others were defined as mis-oriented bundles. In addition, flattened and misshapen bundles and loss of stereocilia were also defined as abnormal stereocilia [31]. OHCs displayed a V-shape hair bundle in control mice. However, the stereocilia of OHCs from *Sptan1-*CKO mice lost their polarity and displayed unusual shapes, such as “C” and “S” shapes, or showed a disrupted staircase architecture, such as the fusion of stereocilia (Fig. 3a–c). Of note, abnormalities in the stereocilia of OHCs in *Sptan1-*CKO mice increased with age, with loss of stereocilia observed at P15 and P30 (Fig. 3d), and more severe dysmorphologies in apical and middle regions than basal region (Fig. 3a). Additionally, the shapes of the cuticular plates (indicated by blue circles) were also abnormal in some OHCs from P3 (round or oval shapes), and more OHCs presented with abnormal cuticular plates at P30 (Fig. 3c). The incidence of stereociliary abnormalities of OHCs throughout entire length of the cochlear basilar membrane at different ages is significantly higher (*P* < 0.001) in *Sptan1-*CKO versus control mice (Fig. 3d). Previous study reported that TRIOBP was necessary to form mature stereocilia rootlets; it was essential for the biogenesis of rootlets that provided durable flexibility at the taper and mechanical rigidity to the stereocilia bundle. Then, to confirm the abnormal rootlets of stereocilia in *Sptan1-*CKO mice, we further performed IF of TRIOBP at P3, P7, P15, and P30. Mice in the control group had normal V-shaped rootlets in OHCs whereas in *Sptan1-*CKO mice, rootlets were misoriented from P3 (Fig. 4a–d), and some even lost from P15 (Fig. 4c–d). In line with these results, while the rootlets of IHCs stereocilia were not lost until P30, there were slightly disordered (Fig. 4d).

We further investigated the changes in HC morphology using myosin VIIa, a specific marker of HCs. The morphology of most OHCs became irregular, such as round or polygons shapes in *Sptan1-*CKO mice at P15 (Fig. S3a), an alteration that was further aggravated at P30. In addition, a massive loss of OHCs occurred at P30; the surviving cells were those in which the Cre recombinase was too weak to knockout SPTAN1 (Fig. S3b).

### The Abundance of Hair Cells Is Decreased in *Sptan1*-CKO Mice Due to Apoptosis

**Fig. 5:**
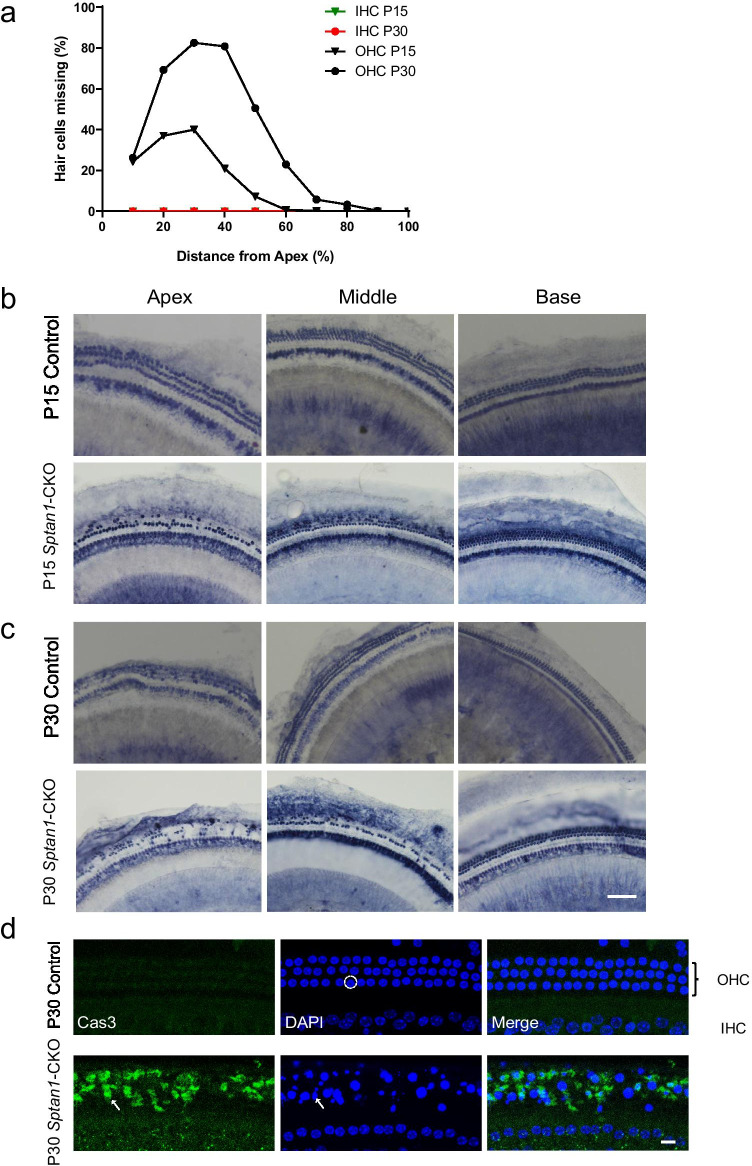
Loss of hair cells in *Sptan1*-CKO mice at different ages. **a** At P15, *Sptan1*-CKO mice show a loss of up to 40% of outer hair cells (OHCs) at the apical-middle turns, but not at the middle-basal turns. At P30, more severe OHC loss is observed in *Sptan1*-CKO mice, with no inner hair cells (IHCs) loss. **b** Succinate dehydrogenase histochemistry (SDH) staining of hair cells in *Sptan1*-CKO mice at P15. **c** SDH staining of hair cells showing OHCs loss in *Sptan1*-CKO mice at P30. Scale bar: 25 µm. **d** At P30, no caspase-3-positive OHCs and IHCs are observed in control mice. The large and round nuclei (blue) of normal hair cells in control mice are indicated by circles. However, OHCs from *Sptan1*-CKO mice show the presence of caspase-3 activation (green) and shrunken and fragmented nuclei, as highlighted by the arrows. The middle turns of the cochlea were used in **d**. Scale bar: 10 µm

To determine whether *Sptan1* deficiency affected HC abundance, HCs were counted using Myosin 6 in *Sptan1*-CKO and control mice at P3, P7, P15, and P30. No HCs were lost at P3 and P7, while OHCs were apparently lost at P15 and P30 (Fig. S4). Next, we used succinate dehydrogenase histochemistry (SDH) to quantitatively evaluate the HC loss in both groups at P15 and P30. The mean cochleograms (*n* = 6 in each group at each stage) indicated that OHCs were rapidly and progressively lost in *Sptan1*-CKO mice. At P15, up to 40% of OHCs were lost at the apical-middle turns, whereas a few were lost in the middle-basal turns. At P30, more severe OHC loss occurred, with approximately 80% of OHCs loss at the apical-middle turns and 5% loss at the middle-basal turns (Fig. 5a–c). Of note, the IHC abundance was unaffected at P15 and P30 (Fig. 5a–c, S2-3). Similar results were observed in the P60 mouse cochlea, with the majority of OHCs absent in the apical-middle turns (Fig. S5).

We then investigated whether OHCs underwent programmed cell death. HCs were labeled with DAPI for the visualization of the nuclei at P30. The HCs of control mice exhibited characteristic cell nuclei shapes, i.e., large and round nuclei (Fig. 5d), whereas the nuclei of *Sptan1*-CKO mice were darkly stained and severely shrunken, or fragmented (Fig. 5d). Furthermore, cochleae were labeled with cell-permeable fluorogenic probes specific to caspase-3 to determine if the programmed cell death was caspase-mediated. Caspase-3 was not activated in control mice, whereas positive caspase-3 labeling was observed in *Sptan1*-CKO mice (Fig. 5d).

### The Cuticular Plates of *Sptan1*-CKO Mice Show Decreased/Absent SPTBN1 Expression

**Fig. 6:**
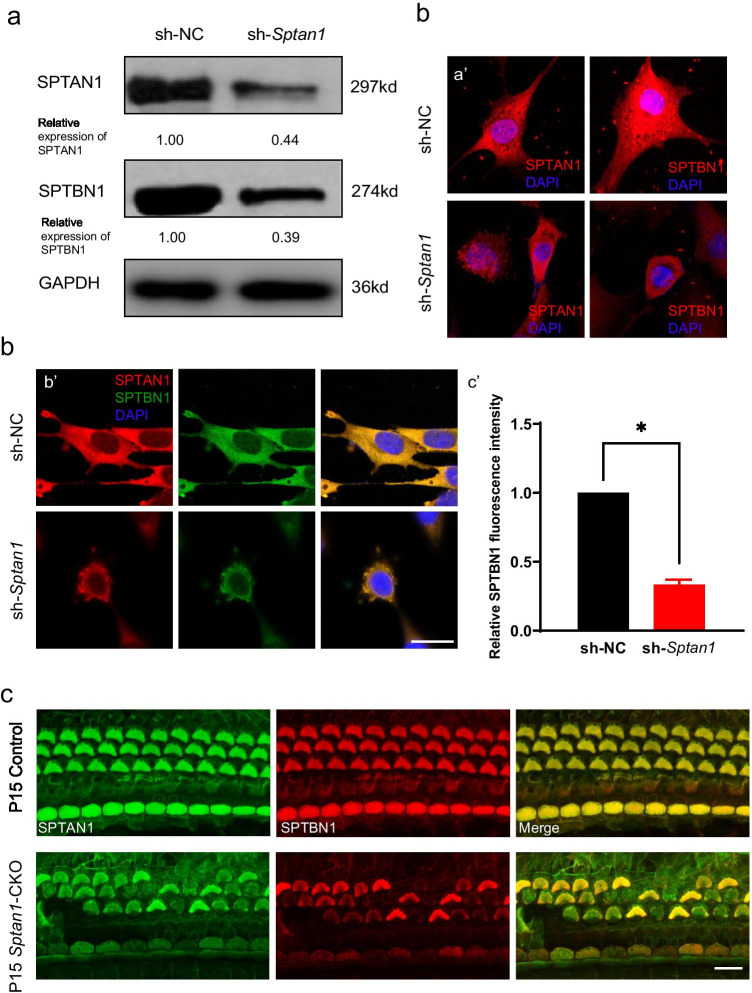
SPTBN1 is decreased or absent in *Sptan1* knockdown HEI-OC1 cells and in HCs from *Sptan1*-CKO mice. **a** Representative western blotting showing the protein levels of SPTAN1 and SPTBN1. The SPTAN1 and SPTBN1 levels are decreased in *Sptan1-*depleted (sh-*Sptan1*) versus control (sh-NC) cells. GAPDH was used as the loading control. **b** (a′–b′) Cells in the sh-*Sptan1* show decreased SPTAN1 and SPTBN1 levels compared to those in sh-NC-treated cells. Scale bar: 10 µm. (c′) Relative quantification of SPTBN1 protein by intensity analysis. Data are shown by means ± SEM. * *P* < 0.05. **c** In *Sptan1*-CKO mice, the absence of SPTAN1 is associated with the absence of SPTBN1 in cochlear hair cells at P15. Scale bar: 10 µm

Previous studies have reported that SPTAN1 and SPTBN1 interact with each other in the cuticular plates of cochlear HCs in mice [21]. Therefore, we examined the expression of SPTBN1 in *Sptan1* knockdown HEI-OC1 cells and *Sptan1*-CKO mice. The results indicated that *Sptan1* knockdown led to decreased levels of SPTBN1 in HEI-OC1 cells (Fig. 6a). Furthermore, the expression of SPTBN1 also differed in *Sptan1* depletion (sh-*Sptan1*) group from small hairpin negative control (sh-NC) cells (Fig. 6b). Then, SPTAN1/SPTBN1 immunofluorescence staining was performed with whole-mount basilar membranes at P15. SPTAN1 and SPTBN1 were co-expressed in the cuticular plate with the same distribution in control mice but absent or downregulated (both proteins) in *Sptan1*-CKO mice (Fig. 6c).

### *Sptan1* Plays a Role Through Regulating Focal Adhesion Signaling

**Fig. 7:**
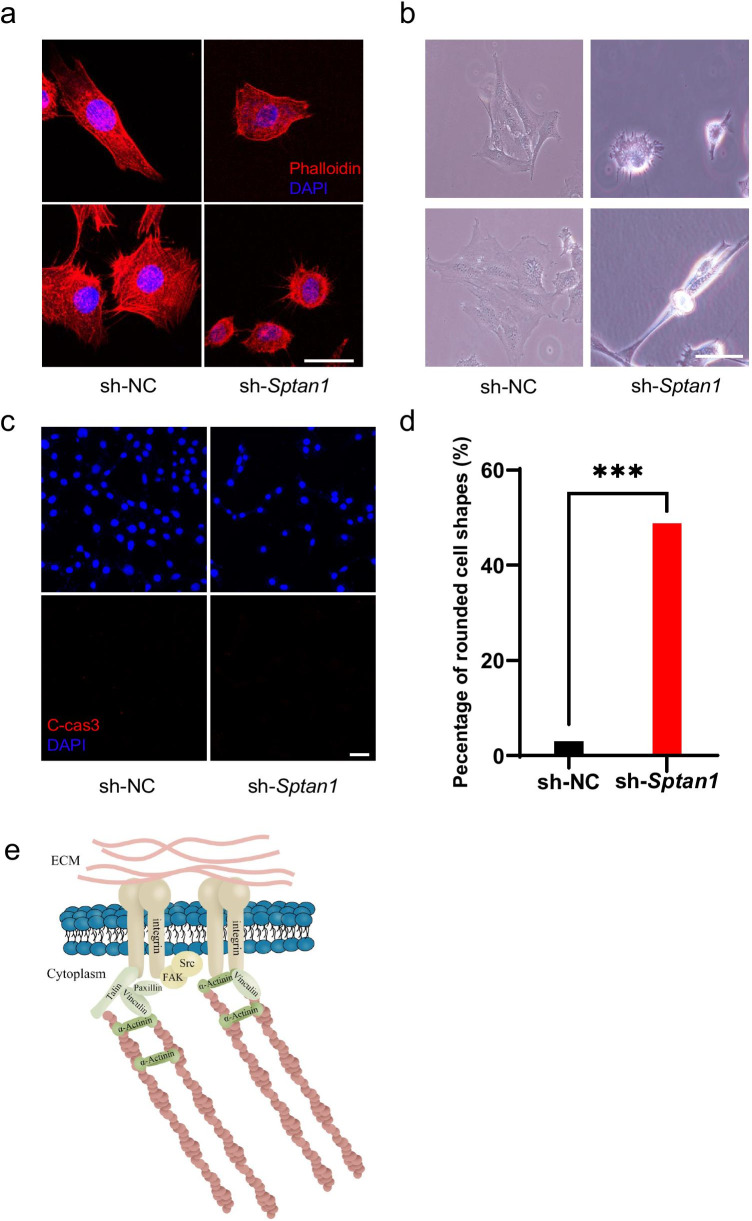

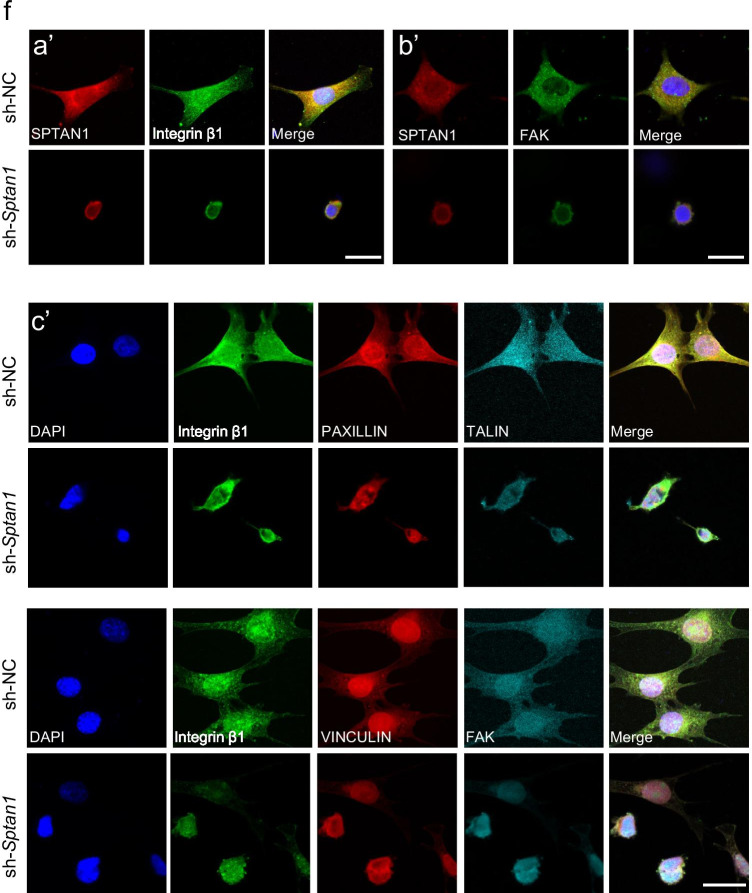
*Sptan1* knockdown HEI-OC1 cells exhibit altered cell shapes and decreased spreading. **a** F-actin organization in HEI-OC1 cells. Phalloidin labeling is intense in the cytoplasm, and stress fiber-like filaments are clearly observed in control (sh-NC) cells. In *Sptan1-*depleted (sh-*Sptan1*) cells, phalloidin labeling is present in both the cytoplasm and cortex of the cell body, with the loss of stress fiber-like filaments. Scale bar: 10 µm. **b** Compared to sh-NC cells, sh-*Sptan1* cells show decreased spreading. Scale bar: 50 µm. **c** The cell viability of sh-*Sptan1* and sh-NC cells is comparable. Scale bar: 50 µm. **d** The percentage of rounded cell shapes in the sh-*Sptan1* group is significantly higher than that in the sh-NC group (~ 350 cells in each group). Error bars represent standard error of the mean. ***P < 0.001 **e** The illustration of proteins involved in focal adhesions. **f** (a′) The co-localization of SPTAN1 and Integrin β1 in sh-NC and sh-*Sptan1* groups. (b′) The co-localization of SPTAN1 and FAK in sh-NC and sh-*Sptan1* groups. (c′) Control cells extend flat membrane protrusions (lamellipodia) and fingerlike protrusions (filopodia) whereas focal adhesions are disrupted in sh-*Sptan1* cells. The components of focal adhesion are gathered around the nucleus. Scale bar: 10 µm

The actin cytoskeleton is critical for the maintenance of cell shape and abundance in the cuticular plate. The altered morphology of the cuticular plate in *Sptan1*-CKO mice prompted us to further examine the changes in cell shape and spreading. F-actin of sh-NC cells was mainly located in the cytoplasm, and stress fiber-like filaments were clearly observed (Fig. 7a). Sh-NC cells also manifested various shapes, including fusiform or polygonal cell shapes, and extended flat membrane protrusions (lamellipodia), and fingerlike protrusions (filopodia) (Fig. 7a–b). However, depletion of *Sptan1* caused cell rounding; a large portion of the cells retracted their protrusions (Fig. 7a–b, and f). Furthermore, marked changes in the actin cytoarchitecture were observed, including the loss of distinct arc-shaped actin bundles (Fig. 7a). Of note, we performed IF of cleaved-caspase 3 to confirm that the abnormal shape of sh-*Sptan1* cells was not due to the loss of viability (Fig. 7c) and quantitatively analyzed the percentage of rounded cell shapes in two groups. In line with the above results, this quantitative analysis revealed that the number of rounded cells in the sh-*Sptan1* group was significantly higher (*P* < 0.001) than that in the sh-NC group (Fig. 7d). In addition, we analyzed the proteins that may interact with SPTAN1 and SPTBN1 using mass spectrometry analysis. We found that SPTAN1 co-precipitated with SPTBN1, and SPTBN1 co-precipitated with Integrin β1, TALIN-1, and VINCULIN (Table. S4). Cell adhesion and spreading require integrin-mediated formation of focal adhesions, and the structural modifications of focal adhesions require the assistance of focal adhesion kinase (FAK), PAXILLIN, VINCULIN, and TALIN to mediate the interaction between the extracellular matrix (ECM) and the actin cytoskeleton (Fig. 7e). Integrins promote the formation of signaling complexes that regulate F-actin accumulation, which is best characterized by focal adhesions containing FAK, and it has been reported that FAK was located at the apical HC surface and in stereocilia. Therefore, we further examined whether the depletion of *Sptan1* caused its inability to bind to focal adhesion-related proteins, which led to abnormal actin cytoskeletal structure and decreased cell spreading in the sh-*Sptan1* group. Here, we studied expression of proteins including FAK, Integrin β1, PAXILLIN, TALIN, and VINCULIN in sh-NC and sh-*Sptan1* groups. Integrin β1 and FAK were co-localized with SPTAN1 (Fig. 7f (a′–b′)). In sh-NC group, these proteins were expressed in nucleus and cytoplasm, whereas in the sh-*Sptan1* group, morphology of these focal adhesions-related proteins was disrupted, which led to destruction of formation and maintenance of cytoskeletal architecture including stress fibers (Fig. 7f (c′)).

**Fig. 8:**
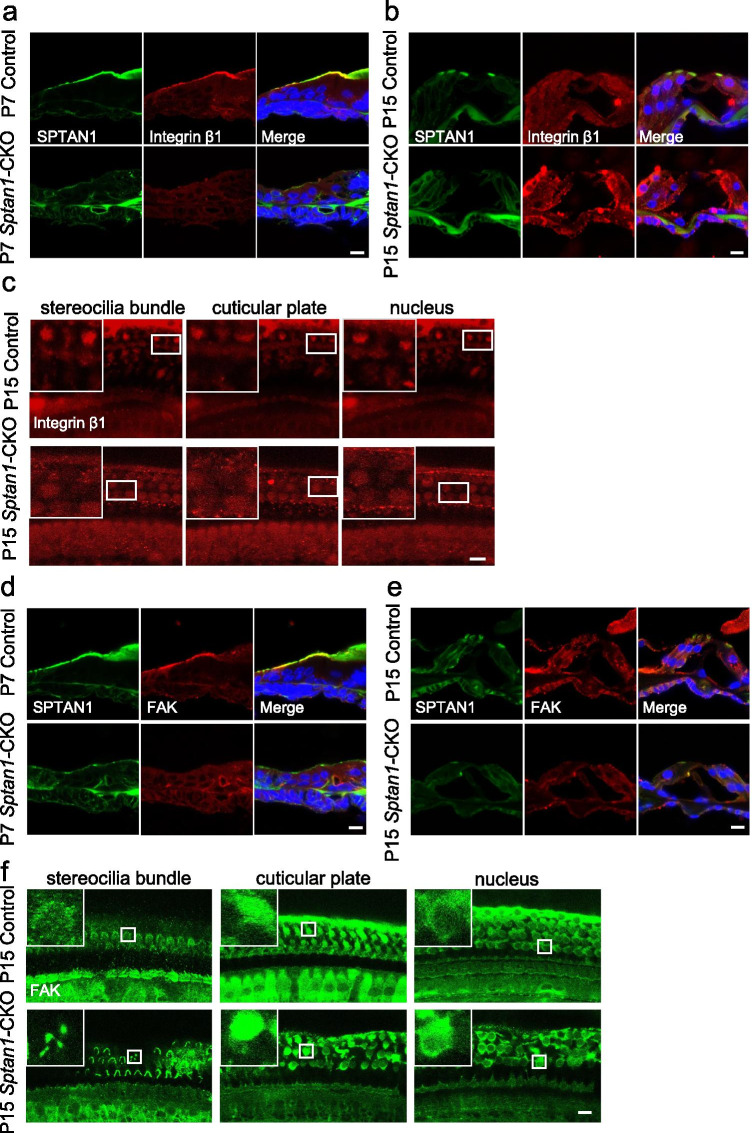

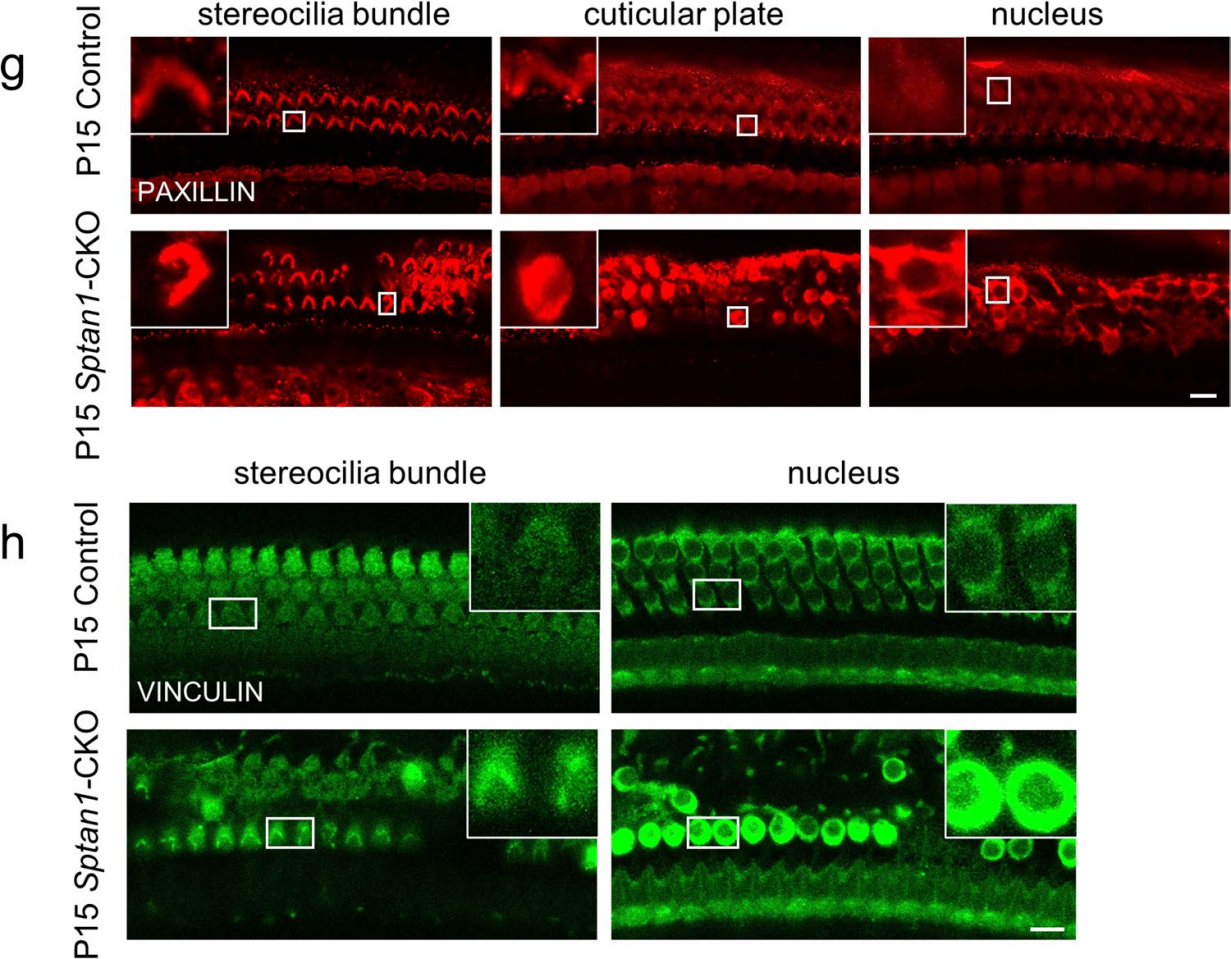
The loss of *Sptan1* affects focal adhesion signaling in P7 and P15 mice. **a** Cryosections showing the absence of Integrin β1 from the cuticular plate in P7 *Sptan1*-CKO mice. **b** Cryosections showing the absence of Integrin β1 from the cuticular plate in P15 *Sptan1*-CKO mice. **c** Whole-mount staining showing a decreased fluorescence intensity of Integrin β1 in P15 *Sptan1*-CKO mice. **d** Cryosections showing the absence of FAK from the cuticular plate in P7 *Sptan1*-CKO mice. **e** Cryosections showing the absence of FAK from the cuticular plate in P15 *Sptan1*-CKO mice. **f** Whole-mount staining showing the abnormal aggregation and distribution of FAK in P15 *Sptan1*-CKO mice. **g** Whole-mount staining showing the abnormal aggregation and distribution of PAXILLIN in P15 *Sptan1*-CKO mice. **h** Whole-mount staining showing the abnormal aggregation and distribution of VINCULIN in P15 *Sptan1*-CKO mice. The middle turns of the cochlea were used. c and f–h represent the expression of proteins in different focal planes including stereocilia bundle, cuticular plate, and nucleus of the hair cells. Scale bar: 10 µm

Furthermore, for the sake of validation, we examined the formation of integrin-focal adhesions in P7 and P15 mice. In *Sptan1*-CKO mice, Integrin β1 and FAK were absent in the cuticular plate at P7 (Fig. 8a, d) and P15 (Fig. 8b–c, e–f). The absence of Integrin β1 and FAK in the cuticular plates occurred at P7, and the HCs were not missing at this time (nucleus still existed) (Fig. 8a, d). Therefore, we have reached the conclusion that reduced Integrin β1 and FAK in the cuticular plates were related to the knockout of *Sptan1*, but not because of missing of HCs. Additionally, the abnormal aggregation or absence of PAXILLIN (Fig. 8g) and VINCULIN (Fig. 8h) at P15 were also observed. Therefore, we concluded that the knockout of *Sptan1* led to the absence of integrin-focal adhesion signaling, and it might indirectly bind to focal adhesion-related proteins by binding to SPTBN1 to play the role in hair cell morphology and function.

## Discussion

Together, using a HC-specific *Sptan1* knockout mouse model, we found that *Sptan1* is essential for maintaining stereocilia and cuticular plate morphologies. *Sptan1*-CKO mice presented with rapid OHC loss and early-onset deafness. Additionally, shRNA-mediated *Sptan1* knockdown in HEI-OC1 cells led to abnormal actin distribution and decreased cell spreading. Importantly, the deficiency of *Sptan1* induced the abnormal formation of focal adhesions and integrin signaling in both HEI-OC1 cells and mouse HCs. In summary, the results of these in vivo and in vitro studies suggest that *Sptan1* is indispensable for the maintenance of normal cuticular plate and stereocilia and the consequent survival of cochlear HCs via focal adhesion signaling.

In HCs, SPTAN1 was expressed in cuticular plate, rootlets of stereocilia, and along the lateral wall of HCs. In addition, it was still expressed in the cortical lattices of Deiters’ cells, pillar cells, and other supporting cells. The expression changes detected by RT-PCR and western blotting could also reflect supporting cells changes in part. Previous study reported that in the cochlea of mouse, rootlets began to develop around E15, and the cuticular plate then forms around the rootlet by E18 [32]. Another study observed rootlets development in mice from P1, with the upper rootlet appearing first and then extending down into the cuticular plate [33]. The SPTAN1 was abundantly expressed in the early postnatal stages, which supported the idea that SPTAN1 in the HC is essential for rootlets and cuticular plates development. In addition, although it started to decrease at around P7, it was still abundantly expressed at P15 and P30, which suggested that it may play an essential role in maintaining the morphology of stereocilia and cuticular plate during late postnatal stages. As global *Sptan1* knockout mice are embryonic lethal [34], we used the *Gfi1-Cre* mouse line as a tool to generate HC-specific *Sptan1* deficient mice, to define the roles of *Sptan1* in cochlear HCs. We found some degree of mosaicism in the context of the *Gfi1-Cre*^*−/*+^-mediated recombination in HCs in the context of different ages, likely due to the low expression level of *Cre* in those cells. Of note *Gfi1-Cre*^*−/*+^;*Sptan1*^*f/f*^ mice presented with decreased expression levels of *Sptan1* in most IHCs and OHCs at birth as well as a progressive loss of this protein with age. Previously, the recombination efficiency of *Gfi1-Cre* in cochlear hair cells was shown to be approximately 93% [24], and our study showed comparable rates of approximately 81%. Importantly, we cannot exclude the possibility that the timing of *Gfi1-Cre* expression is slightly different in different types of hair cells, which may account for the differential effective rates [35]. In spite of the different efficiency in OHCs with a decreased or absent expression of *Sptan1*, the stereocilia and cuticular plates lost their normal appearance. Although that study reported that heterozygous *Gf1*^*Cre*^ mice also exhibited early-onset progressive hearing loss, auditory thresholds were not significantly affected, at least in the range of 8 to 16 kHz for up to 3 months after birth. In contrast, in our study, *Sptan1*-CKO mice showed a low-frequency hearing loss, suggesting that this animal model is suitable for studying the correlation between *Sptan1* expression and changes in cochlear HC morphology and auditory function. In our study, hearing loss was assessed in far-field recording using ABRs, and in acute, near-field recordings of the CAP. DPOAE was used to assess the function of OHCs. The thresholds of ABR and CAP were slightly different (maybe due to operations differences); however, the results all showed that hearing thresholds at different frequencies were elevated in *Sptan1*-CKO group compared to the control group. They all proved that the *Sptan1*-CKO mice developed hearing loss, and these were in consistent with the morphological evidence.

*Sptan1* deficiency in OHCs of *Sptan1*-CKO mice was associated with abnormal stereocilia and cuticular plate morphology. The cuticular plate is enriched with F-actin [7]. *Sptan1* deficiency led to flattened morphological changes in the cuticular plates, which may affect the distribution and state of F-actin. Therefore, we performed in vitro studies via the shRNA-mediated *Sptan1* knockdown in HEI-OC1 cells. Interestingly, *Sptan1* knockdown HEI-OC1 cells showed shrunken sizes, disorganized F-actin, and loss of stress fiber-like filaments, indicating that *Sptan1* is crucial for the maintenance of the shape of cultured HEI-OC1 cells through collaboration with F-actin. In addition, the deletion of *Sptan1* led to the decreased expression of SPTBN1. Previous study reported that spectrins function as tetramers that consist of two α and two β subunits [36], and the spectrin tetramers line the internal side of the plasma membrane, where they interact with integral membrane proteins and F-actin [37]. In cochlear HCs, SPTAN1 and SPTBN1 form a cylindrical structure in the rootlet region of each stereocilium, probably important for the shearing of mechanical stress during deflection [21]. In addition, the results of mass spectrometry analysis showed that SPTAN1 co-precipitated with SPTBN1, which suggested that SPTAN1 and SPTBN1 co-localized in the rootlets of stereocilia and cuticular plates and bound to each other to stabilize the stereocilia. HC-specific *Sptbn1* knockout mice displayed severe hearing loss and exhibited more severe phenotypes in the context of OHC stereocilia compared to that of IHC stereocilia [21], which was consistent with the phenotypes of HC-specific *Sptan1* knockout mice. Moreover, Liu and colleagues reported that SPTAN1 was still found specifically in the cortical lattice region of HCs in *Sptbn1* knockout mice [21], whereas SPTBN1 was absent in *Sptan1*-deficient cochlear HCs and HEI-OC1 cells in our study. Therefore, altogether, our results suggest that SPTAN1 may be required for the specific localization and function of SPTBN1 in the cuticular plate of HCs; SPTAN1 is likely an essential component of the direct or indirect maintenance of stereocilia. In addition, compared with other regions, the low-frequency region was affected more severely in *Sptan1*-CKO mice. This may be due to the higher efficiency of *Gfi1-Cre* recombinase-mediated recombination in the apical turns, more HCs lacked the expression of *Sptan1*, which led to more HCs loss. The difference further proved that *Sptan1* was essential for cochlea hair cell morphology and function. However, the specific mechanism behind this particular phenotype needs to be further studied.

In addition to anchoring stereocilia directly at the rootlet, our findings suggest that *Sptan1* plays a role in the maintenance of the morphology and function of stereocilia and HCs via the regulation of integrin-focal adhesion signaling. Integrin-tethered focal adhesions participate in mechanosensing, structure, and signaling [38]. Integrins can interact with the ECM to provide cells with support and survival signals [39]. Mutations in ECM components, such as type IV collagen, can lead to hereditary hearing loss, and integrins may be involved in this process. Additionally, sensory HCs have an elaborate F-actin cytoskeleton, and integrins can regulate the dynamics of actin. In fact, a previous study reported that α8β1 integrin are proteins that are required for normal hearing and stereocilia structure, stereocilia did not achieve complete maturation or were not maintained after the knockout of integrin α8 [40]. Another study demonstrated that integrin α8 and protocadherin-15 act as a complex to regulate cilia biogenesis in sensory cells [41]. Therefore, we speculate that *Sptan1* might interact with Integrin β1 to maintain the stereocilia in the cuticular plate. Of note, integrins promote the formation of signaling complexes that regulate F-actin accumulation, which is best characterized by focal adhesions containing FAK [42]. FAK is a key component of integrin activated signaling pathways that is recruited to the apical hair cell surface during stereocilia formation [40, 43]. Previous study reported that in the noise exposed organ of Corti, FAK p-Tyr577 was detected in OHC stereocilia of noise damaged regions. Excessive noise-induced mechanical stress of stereocilia induced FAK phosphorylation in stereocilia [44]. These data indicate that α8β1 integrin and FAK are in a molecular pathway that regulates the assembly or maintenance of the cytoskeleton of the stereocilia. Another study reported that TALIN was expressed in anchoring cells that could create and/or maintain tension on the spiral ligament-basilar membrane complex and influence the mechanical properties of the basilar membrane [45]. Importantly, our results suggested that SPTAN1 indirectly interacted with focal adhesion-related proteins, and the knockout of *Sptan1* caused the abnormal aggregation or absence of FAK, PAXILLIN, TALIN, and VINCULIN. Therefore, we conclude that *Sptan1* may affect actin dynamics through focal adhesion signaling, resulting in stereocilia formation and maturation disorders. Of note, focal adhesions had not actually been observed in our study so the link remains speculative, and more experimental verification should be carried out to explore the mechanisms that govern both the direct and indirect interactions between *Sptan1* and the components of the signaling complexes.

The rapid death of HCs is secondary to *Sptan1* deficiency. Defects in any of the sensory cells of the organ of Corti including IHCs, OHCs, the tectorial membrane, supporting cells, or cells of the stria vascularis can lead to deafness. In our study, loss of OHCs is the major cochlear pathologies in early-onset hearing loss. *Sptan1*-CKO mice demonstrated rapidly progressive OHC loss starting from the apex, with the most severe OHC loss at the apical-middle turns observed as early as P15; additionally, IHC loss was observed until P60. Damage patterns progressing from partial to complete OHCs lesioning and eventually to the IHCs. The different fates of both types of HCs were reported in previous studies [33, 46]. The OHCs are one of the most vulnerable components in most pathologies [47, 48]. In our study, though the expression of SPTAN1 in IHCs and OHCs appeared similar, the loss of OHCs was more sever. Possible reasons of different fates IHCs and OHCs in our study might include the following. Previous study reported that the cortical network involved in the sound-induced electromotility of OHCs contains SPTAN1 [18], and we speculated that the depletion of *Sptan1* may decrease the electromotility of OHCs. In addition, we cannot definitively exclude the presence of unknown partners, exclusively expressed in IHCs and OHCs that modulate *Sptan1* or undergo other regulations. A difference in actin or microtubule metabolism in OHCs versus IHCs might also explain the difference of phenotype between the two HCs. Furthermore, *Sptan1* is also involved in diverse processes occurring in the nucleus, and the loss of *Sptan1* in HCs could have led to chromosome instability [49]. *Sptan1* is speculated to be a major substrate for calpain and caspase-3 cysteine proteases [50]. In addition, *Sptan1*-CKO mice may also undergo necroptosis and necrosis, but we did not detect them. Therefore, the cell death-related pathway affected after *Sptan1* knockout remains to be determined.

In conclusion, our findings suggested that the Sptan1-CKO mice displayed abnormal OHC and IHC stereocilia morphology, including improper bundle orientation, and the cuticular plate lacked its characteristic oval shape, which was more pronounced in OHCs. After hearing onset (P12), the OHC stereocilia showed rapid degeneration, followed by HCs loss proceeding from apex to base. Sptan1 depletion may affect the formation of integrin-focal adhesions, leading to abnormal actin cytoskeletal structure and decreased cell spreading. Therefore, our study indicated that Sptan1 is essential for the cochlear hair cell morphology and function, which plays the role via the regulation of focal adhesion signaling.

## Supplementary Information

Below is the link to the electronic supplementary material.

Supplementary file1 (DOCX 30.6 KB)

## Data Availability

The datasets used and/or analyzed during the current study are available from the corresponding author on reasonable request.
