# Supplementary material for: Essential Role of Sptan1 in Cochlear Hair Cell Morphology and Function Via Focal Adhesion Signaling
Source: Mol Neurobiol. 2021 Oct 27;59(1):386–404. doi: 10.1007/s12035-021-02551-2 (PMC8786805; doi:10.1007/s12035-021-02551-2)
Supplement: Supplementary file 1 — Supplementary file1 (DOCX 30.6 KB) [file 12035_2021_2551_MOESM1_ESM.docx]

Essential role of *Sptan1* in cochlear hair cell morphology and function via focal adhesion signaling

Qingxiu Yao^1,2,3#^, Hui Wang^4#^, Hengchao Chen^5#^, Zhuangzhuang Li^1,2,3^, Yumeng Jiang^1,2,3^, Zhipeng Li^1,2,3^, Jiping Wang^1,2,3^, Yazhi Xing^1,2,3^, Feng Liu^1,2,3,^*, Dongzhen Yu^1,2,3,^*, Shankai Yin^1,2,3^

^1^Department of Otolaryngology-Head and Neck Surgery, Shanghai Jiao Tong University Affiliated Sixth People's Hospital, Shanghai, 200233, China;

^2^Otolaryngology Institute of Shanghai Jiao Tong University, Shanghai, 200233, China;

^3^Shanghai Key Laboratory of Sleep Disordered Breathing, Shanghai, 200233, China;

^4^ENT Institute and Otorhinolaryngology Department, Affiliated Eye and ENT Hospital, Fudan University, Shanghai, 200031, China;

^5^The First Affiliated Hospital, Zhejiang University School of Medicine, Hangzhou, 310003, China;

**^#^**These authors contributed equally to this work

*** Corresponding author:** Dongzhen Yu, Feng Liu
E-mail address: [drdzyu@126.com](mailto:drdzyu@126.com), [liufeng@sibs.ac.cn](mailto:liufeng@sibs.ac.cn)

ORCID: https://orcid.org/0000-0001-9424-4855

**Supplemental Figure Legends**

**Fig. S1** Generation of a conditional *Sptan1* knockout mouse model. **(a)** Schematic representation of the alleles of the *Sptan1* gene in wild-type mice. LoxP sites were added on both sides of 6, 7, 8 exons. **(b)** The primer locations in *Sptan1*-CKO mice. **(c)** PCR analysis of control and *Sptan1*-CKO mice with different primers

**Fig. S2** The expression of SPTAN1. (a) The expression of SPTAN1 was roughly equal expressed at apical, middle and basal turns at P1, P3, P7, and P30. (b) SPTAN1 was expressed in cuticular plates and along the lateral wall of hair cells, and the cortical lattices of Deiters' cells and pillar cells. Scale bar: 10 μm

**Fig. S3** Loss of *Sptan1* affects the shapes of OHCs. **(a)** Maximum intensity projections of confocal Z-stacks of the *Sptan1*-CKO and control organs of Corti immunostained with myosin VIIa antibody (red) and SPTAN1 (green). At P15, most cuticular plates changed to an oval shape. **(b)** At P30, most OHCs were missing, the surviving cells were probably those in which the Cre recombinase was too weak to knockout SPTAN1. And the surviving OHCs had irregular cuticular plates that were nearly round. The abnormal cuticular plate is indicated by arrows. Scale bars: 10 µm

**Fig. S4** *Sptan1* deficiency affected hair cells (HCs) abundance at different ages. HCs were counted using Myosin 6 at P3, P7, P15, and P30 in two groups. No HCs loss was observed at P3 and P7, while outer hair cell loss at P15 and P30 was apparent. Images of middle turns of the cochleae were captured. Scale bar: 10 µm

**Fig S5** The missing hair cells at P60. There were more outer hair cells missing at P60 than at P30. Some inner hair cells (IHCs) were also lost, as indicated by arrows. The apical turn had approximately 30% of IHCs missing, and the middle and basal turns had less IHC loss. Scale bar: 50 µm

# Supplemental Tables

**Table. S1** Primers used for *Sptan1* and *Gfi1-Cre* identification

| Target | Primer sequence |
| --- | --- |
| *Sptan1* f | 5′-GATGCTCAATCTGACCTGCA -3′ |
| *Sptan1* r | 5′-CATCAATGCACTCCCATCTA-3′ |
| *Sptan1* 3’ | 5′-GCATCGCATTGTCTGAGTAGGTG-3′ |
| *Sptan1* 3’ | 5′-CAGCACCAGCCACATCATTG-3′ |
| *Sptan1* 5’ | 5′-GTTCTGACTAGGTGTAAGTAT-3′ |
| *Sptan1* 5’ | 5′-AAGGGTTATTGAATATGATCGGA-3′ |
| Gfi1Cre-R | 5′-GCCCAAATGTTGCTGGATAGT-3′ |
| Gfi-1F | 5′-GGGATAACGGACCAGTTG-3′ |
| Gfi-1R | 5′- CCGAGGGGCGTTAGGATA-3′ |

**Table. S2** Primers used for qRT-PCR

| Target | Primer sequence |
| --- | --- |
| *Sptan1* RT forward | 5’-GCTAACTCAGGAGCCATTG-3’ |
| *Sptan1* RT reverse | 5’-AGTGCCCTTCAGAAATCATC-3’ |
| GAPDH RT forward | 5’-TCATCCCAGAGCTGAACG-3’ |
| GAPDH RT reverse | 5’-TCATACTTGGCAGGTTTCTCC-3’ |

**Table. S3** Antibodies used in Immunofluorescence staining

|  |  | Purpose |
| --- | --- | --- |
| Primary antibody | Alpha fodrin (Abcam) | Against SPTAN1. *Sptan1* encodes spectrin αⅡ (Alpha fodrin). |
|  | SPTBN1 (BD Biosciences) | Against SPTBN1. |
|  | Myosin VIIa (Proteus BioSciences) | A marker of cochlear hair cell, to study morphology of hair cells. |
|  | Myosin 6 (ProteinTech Group) | To visualize hair cell abundance. |
|  | TRIOBP (ProteinTech Group) | To visualize the rootlet of stereocilia. |
|  | Fak (ProteinTech Group) | To visualize formation of focal adhesion. |
|  | Integrin β1 (ProteinTech Group) | To visualize formation of focal adhesion. |
|  | Talin (ProteinTech Group) | To visualize formation of focal adhesion. |
|  | Vinculin (ProteinTech Group) | To visualize formation of focal adhesion. |
|  | Paxillin (ProteinTech Group) | To visualize formation of focal adhesion. |
|  | Cleaved-caspase 3 (Cell Signaling Technology) | To assess apoptosis. |
| Secondary antibody | Alexa Fluor® 488 goat anti-mouse IgG1 (γ1) (Invitrogen) | |
|  | Alexa Fluor® 647 goat anti-mouse IgG2b (γ2b) (Invitrogen) | |
|  | Alexa Fluor® 633 goat anti-mouse (Invitrogen) | |
|  | Alexa Fluor® 633 goat anti-rabbit (Abcam) | |

**Table. S4** Proteins enriched in SPTAN1 and SPTBN1 pellets

| SPTAN1 | SPTBN1 |
| --- | --- |
| Serum albumin | Serum albumin |
| Serotransferrin | SPTAN1 |
| Actin, cytoplasmic 1 | Actin, cytoplasmic 1 |
| SPTBN1 | VINCULIN |
| Annexin A5 | Integrin beta-1 |
| Tubulin alpha-4A chain | TALIN-1 |
